# Engineering atomic-level complexity in high-entropy and complex concentrated alloys

**DOI:** 10.1038/s41467-019-10012-7

**Published:** 2019-05-07

**Authors:** Hyun Seok Oh, Sang Jun Kim, Khorgolkhuu Odbadrakh, Wook Ha Ryu, Kook Noh Yoon, Sai Mu, Fritz Körmann, Yuji Ikeda, Cemal Cem Tasan, Dierk Raabe, Takeshi Egami, Eun Soo Park

**Affiliations:** 1 Department of Materials Science and Engineering, Seoul National University, 1 Gwanak-ro, Gwanak-gu, 08826 Seoul, Republic of Korea; 2 Joint Institute for Computational Sciences, University of Tennessee and Oak Ridge National Laboratory, Oak Ridge, TN 37996 USA; 3 National University of Mongolia, Ulaanbaatar, 14201 Mongolia; 4 Oak Ridge National Laboratory, Oak Ridge, TN 37831 USA; 5 Max-Planck-Institut für Eisenforschung, Max-Planck-Straße 1, 40237 Düsseldorf, Germany; 6 Materials Science and Engineering, Delft University of Technology, 2628 CD Delft, Netherlands; 7 Department of Materials Science and Engineering, Massachusetts Institute of Technology, 77 Massachusetts Avenue, Cambridge, MA 02139 USA; 8 Department of Materials Science and Engineering and Department of Physics and Astronomy, University of Tennessee, Knoxville, TN 37996 USA

**Keywords:** Mechanical properties, Metals and alloys

## Abstract

Quantitative and well-targeted design of modern alloys is extremely challenging due to their immense compositional space. When considering only 50 elements for compositional blending the number of possible alloys is practically infinite, as is the associated unexplored property realm. In this paper, we present a simple property-targeted quantitative design approach for atomic-level complexity in complex concentrated and high-entropy alloys, based on quantum-mechanically derived atomic-level pressure approximation. It allows identification of the best suited element mix for high solid-solution strengthening using the simple electronegativity difference among the constituent elements. This approach can be used for designing alloys with customized properties, such as a simple binary NiV solid solution whose yield strength exceeds that of the Cantor high-entropy alloy by nearly a factor of two. This study provides general design rules that enable effective utilization of atomic level information to reduce the immense degrees of freedom in compositional space without sacrificing physics-related plausibility.

## Abstract (web summary)

Designing complex concentrated alloys with targeted properties for high performance remains challenging because of their complex local atomic environments. Here, the authors show how to engineer atomic-level pressure to customize complexity-induced properties such as solid-solution strengthening.

## Introduction

**Fig. 1:**
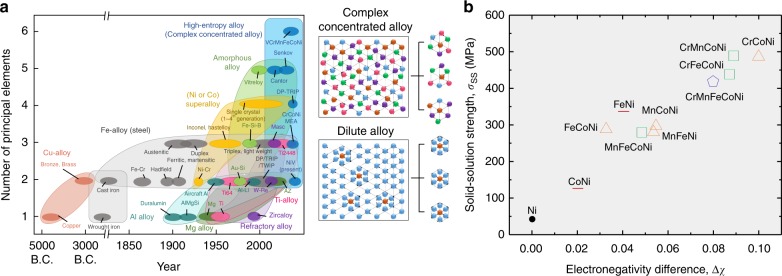
Proportional relationship between electronegativity difference and solid-solution strength in complex concentrated and high-entropy alloys. **a** A historical sketch showing an upward trend in the number of principal elements (≥5 at.%) constituting general alloy systems over the past centuries. It includes Cu-, Fe-, Al-, Mg-, Ti-, and refractory alloys as well as superalloys (Ni-based or Co-based), amorphous and high-entropy alloys. **b** Electronegativity differences among the constituting elements Δ*χ* versus experimentally measured solid-solution strengths *σ*_SS_ of 3d CCAs^25^ for solid-solution strengthening relation. The experimentally measured solid-solution strengths are listed in Supplementary Table 1

For millennia metallurgists have explored mixtures of metallic and non-metallic elements, so-called “alloys”, to produce materials with improved properties. Over the last century, the compositional complexity of alloys has dramatically increased in response to accelerating demands for component safety, efficiency, and resistance to harsh environments (Fig. 1a). Typical examples range from advanced automotive steels to the recently proposed complex concentrated alloys (CCAs) with multi-principal elements at high concentrations^1–3^. The immense composition space compels the design of a practically infinite number of alloys, causing the property-targeted design of high-performance alloys to be extremely challenging. However, the local atomic environments in such alloys are chemically complex, and hence, not amenable to well established methods such as mean-field averaging. The advantage of many degrees of freedom is severely hindered by a lack of quantitative mixing rules, rendering alloy design an empirical trial-and-error undertaking.

In this paper, we present a simple property-targeted quantitative design approach for CCAs based on quantum-mechanically derived atomic-level pressure approximation. This approach is inspired by the effective description of the complex atomic nature of amorphous alloys using atomic-level pressure, which links the elemental information and the local atomic structure to various macroscopic properties such as glass transition and mechanical failure^4^. We show that the dominant factor for solid-solution strengthening in single phase face-centered cubic (fcc) CCAs consisting of 3d transition metal elements (V, Cr, Mn, Fe, Co, and Ni) (3d CCAs) is the variation in the atomic-level pressures originating from the charge transfer between neighboring elements. The material class includes large sub-groups of important commercial alloys such as austenitic steels^5,6^, Ni-based superalloys^7^, and the recently proposed high-entropy alloys (HEAs)^8–11^. We then numerically identify the relationship between configurational difference in the charge transfer and the macroscopic difference in the charge transfer among the elements, which enables connecting the atomic-level fluctuation and complexity to the macroscopic property of the material. It allows establishment of a design recipe which uses elemental information (electronegativity) (Fig. 1b) without explicit electronic structure calculations as an efficient vehicle for more systematic and constitutive structure-property design of CCAs.

## Results

### Atomic-level pressure in complex concentrated alloys

The atomic-level pressure originates from the misfit of an element with its surroundings in terms of its atomic size, electronic state and charge transfer (Supplementary Fig. 1). In conventional dilute alloys, most solutes are surrounded by solvent atoms, with little interaction among solutes (Fig. 1a). Thus, previous approaches for treating the bonding and misfit in dilute alloys assume a fixed atomic-level pressure for a solute element^12,13^. On the other hand, the local atomic environment in CCAs is chemically complex (Fig. 1a)^14,15^; thus the atomic-level pressure fluctuates depending on the specific local environment. We therefore apply the quantum-mechanically derived atomic-level pressure approach. This approach allows calculation of the local electronic energy for finite deformations using the density-functional theory (DFT) technique and calculation of the local stress of an individual atom from numerical derivatives of this energy. This approximation was introduced previously^16,17^, where it was calculated using the locally self-consistent multiple scattering (LSMS) method^18,19^. Details of the method are described in the Methods subsection “First-principles calculations of atomic-level pressure”.

**Fig. 2:**
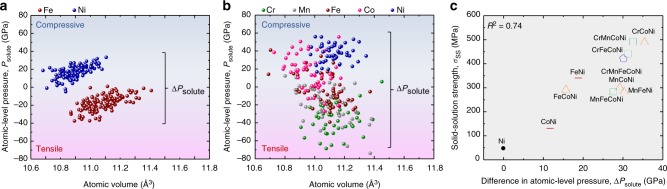
Description of chemical complexity using the difference in atomic-level pressure in 3d CCAs. Atomic-level pressure versus atomic volume relation in **a** the FeNi CCA and **b** the Cantor HEA. A positive value means compressive (repulsive) pressure, and a negative value means tensile (attractive) pressure. **c** Differences in atomic-level pressure $\Delta P_{{\mathrm{solute}}}$ versus experimentally measured solid-solution strengths *σ*_SS_ of 3d CCAs

Figure 2a, b present introductory examples of the DFT calculated atomic-level pressure values for each element in the FeNi CCA (Fig. 2a) and the Cantor HEA (equiatomic fcc CrMnFeCoNi) (Fig. 2b) plotted against the local atomic volume. The volume is defined as the Voronoi-cell volume associated with each atom. Other equiatomic 3d CCAs from the binary CoNi to the quaternary CrFeCoNi are displayed in Supplementary Fig. 2. The model contains 256 atoms in a supercell, and the atoms are randomly mixed without chemical short-range order. Although the net atomic-level pressure, i.e., the sum of all atomic-level pressures, is zero, the individual atoms experience non-zero atomic-level pressure values. Hence, all atoms in CCAs can act as dilatational or compressive sources which interact with defects.

### Solid-solution strengthening versus atomic-level pressure

Solid-solution strengthening is driven by fluctuations in the solute-dislocation interaction energy^20–24^. This energy is caused by the atomic-level pressure through the interaction with the elastic field of a dislocation. As all atoms in the considered 3d CCAs have different pressures, the fluctuation in the interaction energy and the resultant solid-solution strength *σ*_SS_ originate from the configurational fluctuation of the atomic-level pressure^23,24^. If misfit volume in the previous solid-solution strengthening theory of CCAs^23,24^ is replaced by atomic-level pressure *P*_solute_, we obtain the following:

1 $$\sigma _{{\mathrm{SS}}}\propto \left( {\frac{\Gamma }{{b^2}}} \right)^{ - 1/3}\left( {\Delta P_{{\mathrm{solute}}}} \right)^{4/3},$$

where Γ is the line tension, *b* is the Burger’s vector, and Δ*P*_solute_ denotes the ensemble standard deviation of *P*_solute_. In the present 3d CCAs, differences in the moduli (~11%) and the Burger’s vectors (~1%) between the alloys^25^ are negligible compared to the difference in the Δ*P*_solute_ values (~44%). We thus reduce Eq. (1) to $\sigma _{{\mathrm{SS}}}\propto \Delta P_{{\mathrm{solute}}}$ for the sake of simplicity of the parameterization.

Because the Hall-Petch effect is typically invariant with temperature and the Peierls friction stress is very small in fcc materials and is only weakly temperature-dependent, *σ*_SS_ essentially carries the temperature-dependent portion of the yield strength^23,25^. In Fig. 2c, the Δ*P*_solute_ values of 3d CCAs are plotted against the reported temperature-dependent part of the yield strength at 0 K, i.e., solid-solution strength data *σ*_SS_^25^. The proportional relationship clearly reveals that solid-solution strengthening in these alloys indeed originates from the configurational fluctuation of the atomic-level pressure.

### Atomic-level pressure versus charge transfer

**Fig. 3:**
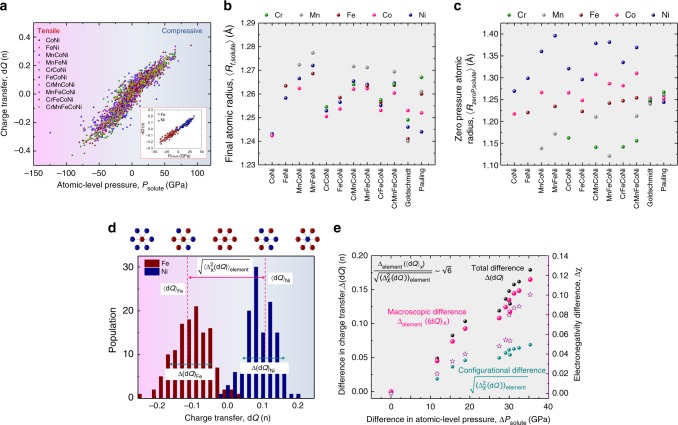
Relationship among atomic-level pressure difference, charge transfer difference and electronegativity difference in 3d CCAs. **a** Relationship between the atomic-level pressure and the charge transfer (change in the number of electrons per atom; A positive value means gaining electrons) of 3d CCAs; inset: FeNi. **b** Average final atomic radii measured by EXAFS and atomic radii of pure elements (Goldschmidt, Pauling). **c** Average zero pressure atomic radii. **d** Charge transfer distribution in FeNi. **e** Relationship among atomic-level pressure difference, charge transfer difference and electronegativity difference in 3d CCAs. Total difference $\Delta \left( {{\mathrm{d}}Q} \right)$ (black dot), macroscopic difference $\Delta _{{\mathrm{element}}}\left( {\left\langle {{\mathrm{d}}Q} \right\rangle _X} \right)$ (pink dot), and configurational difference $\sqrt {\left\langle {\Delta _X^2\left( {{\mathrm{d}}Q} \right)} \right\rangle _{{\mathrm{element}}}}$ (teal dot) in charge transfer, and electronegativity difference (purple star) against the total difference in atomic-level pressure $\Delta P_{{\mathrm{solute}}}$ in 3d CCAs

We now discuss the origin of the atomic-level pressure in 3d CCAs. From the viewpoint of elasticity, the atomic volume for each element is directly linked to its misfit volume and atomic-level pressure^20–22^; large elements should have positive/repulsive pressure and vice versa. Instead, the overall tendency of the present element-averaged atomic-level pressure in Fig. 2a, b does not show any apparent correlation with the atomic volume. Furthermore, the order of atomic-level pressure of each element (Cr<Mn<Fe<Co<Ni) is opposite to the order of atomic sizes of the same elements in pure state (Cr>Mn>Fe>Co>Ni) (Fig. 2b, Supplementary Table 2). On the other hand, Fig. 3a shows a proportional relationship between charge transfer d*Q* that the solute atom experiences and atomic-level pressure in 3d CCAs. This result is a clear indication that the atomic-level pressure (and hence, the misfit volume) of 3d CCAs is driven by the charge transfer, not by the atomic size difference^17^. Thus the relationship between the atomic-level pressure and the charge transfer can be quantified as $P_{s{\mathrm{olute}}} \propto {\mathrm{d}}Q$.

The main reason why the charge transfer is the dominant factor for the atomic-level pressure in the 3d CCAs is that the differences in the atomic sizes among the elements considered here are relatively small. Figure 3b presents the average values of the final atomic radii *R*_f,solute_ measured by extended X-ray absorption fine structure (EXAFS) and the pure atomic radii (Goldschmidt^26^, Pauling^27^) of the constituent elements in the 3d CCAs. We compare these values with the zero pressure atomic radius (Fig. 3c), an imaginary atomic size, before generating atomic-level pressure $R_{{\mathrm{zeroP}},\,{\mathrm{solute}}} = R_{{\mathrm{f}},{\mathrm{solute}}} \cdot \left( {1 + P_{{\mathrm{solute}}}/B_{{\mathrm{solute}},{\mathrm{cluster}}}} \right)^{1/3}$, where $B_{{\mathrm{solute}},{\mathrm{cluster}}}$ is the bulk modulus of a cluster of atoms including the solute; see Supplementary Note 1. The differences among the zero pressure atomic radii are almost one to two orders of magnitude larger than the differences among the pure atomic radii and the differences among the average final atomic radii. This implies significant additional effects on the atomic-level pressure, with the main cause being the charge transfer.

There may be additional reasons for the predominant role of the charge transfer in the atomic-level pressure which originates from the complex nature of CCAs. In metallic liquids and glasses, the local structure (the bond length and the coordination number) effectively changes to accommodate the size misfit effect, and the charge transfer predominantly causes the atomic-level pressure^17,28^. Although CCAs are topologically less complex compared to metallic glasses, fluctuation in local bond lengths^15^ and displacements of atoms from their ideal lattice positions^14^ may accommodate the size misfit effects. This relaxation via the lattice distortion may be a general property of CCAs and further studies are needed.

### Electronegativity difference versus solid-solution strengthening

The significant role of charge transfer in determination of atomic-level pressure demands a departure from the purely mechanical perspective and implies the need of using electronic structure calculations to predict the solid-solution strengthening. The most practical approach to design a new alloy, however, would be a design rule which does not require computationally expensive full-field calculations but instead utilizes specific elemental information that sufficiently represents the underlying atomic information, such as atomic size, electronegativity, and valence electron concentration (VEC)^29^, or experimentally accessible global average atomic properties^30^. Following this idea, we propose a pathway to use such elemental information for predicting fluctuations of the atomic-level pressure in CCAs.

Considering the relation between the atomic-level pressure and charge transfer, the relationship in Eq. (1) can be described by the difference in charge transfer as

2 $$\sigma _{{\mathrm{SS}}} \propto \Delta \left( {{\mathrm{d}}Q} \right).$$

To understand the impact of each chemical element in detail, we decompose $\Delta \left( {{\mathrm{d}}Q} \right)$ as

3 $$\Delta ^2\left( {{\mathrm{d}}Q} \right) = \Delta _{{\mathrm{element}}}^2\left( {\left\langle {{\mathrm{d}}Q} \right\rangle _X} \right) + \left\langle {\Delta _X^2\left( {{\mathrm{d}}Q} \right)} \right\rangle _{{\mathrm{element}}},$$

where $\left\langle {{\mathrm{d}}Q} \right\rangle _X$ is the average of the charge transfers over the atoms of the element *X*, $\Delta _X^2\left( {{\mathrm{d}}Q} \right)$ is the variance of the charge transfers for the element *X*, $\left\langle \cdots \right\rangle _{{\mathrm{element}}}$ is the weighted average of an element-specific quantity over the element, and $\Delta _{{\mathrm{element}}}^2\left( \cdots \right)$ is the weighted variance of element-specific quantity among the constituent elements (see details in Supplementary Note 2). The two terms on the right-hand side reflect two different levels of fluctuations in CCAs. The first term $\Delta _{{\mathrm{element}}}^2\left( {\left\langle {{\mathrm{d}}Q} \right\rangle _X} \right)$ reflects the macroscopic difference in charge transfer among the elements. The second term $\left\langle {\Delta _X^2\left( {{\mathrm{d}}Q} \right)} \right\rangle _{{\mathrm{element}}}$ is the configurational difference in charge transfer caused by different local atomic environments averaged over all constituent elements.

Figure 3d shows an application of this approach in terms of the probability distribution of charge transfers in FeNi in the inset of Fig. 3a. $\Delta _{{\mathrm{element}}}\left( {\left\langle {{\mathrm{d}}Q} \right\rangle _X} \right)$ is 0.093, $\sqrt {\left\langle {\Delta _X^2\left( {{\mathrm{d}}Q} \right)} \right\rangle _{{\mathrm{element}}}}$ is 0.046, and Δ(d*Q*) is 0.104. The ratio between $\Delta _{{\mathrm{element}}}\left( {\left\langle {{\mathrm{d}}Q} \right\rangle _X} \right)$ and $\sqrt {\left\langle {\Delta _X^2\left( {{\mathrm{d}}Q} \right)} \right\rangle _{{\mathrm{element}}}}$ is about 2.0. If we assume that the distributions of Fe and Ni have an isosceles triangle shape and are congruent to each other, the ratio between $\Delta _{{\mathrm{element}}}\left( {\left\langle {{\mathrm{d}}Q} \right\rangle _X} \right)$ and $\sqrt {\left\langle {\Delta _X^2\left( {{\mathrm{d}}Q} \right)} \right\rangle _{{\mathrm{element}}}}$ becomes $\sqrt 6$ (∼2.45). Indeed, the ratios for all considered 3d CCAs were between 2.02 and 2.41 (Fig. 3e and Supplementary Fig. 3). This implies that the stronger the macroscopic difference in charge transfer is, the greater is the difference (fluctuation) of charge transfer from the variation in local atomic environments. This result makes it possible to predict a configurational fluctuation of charge transfer, and hence the atomic-level pressure, using the average charge transfer of each element d*Q*_*X*_, which can be simply approximated by the local electronegativity difference $\chi _X - \chi _{{\mathrm{element}}}$, where *χ*_X_ is the electronegativity of element *X* (Supplementary Table 2), and *χ*_element_ is the weighted average electronegativity over the element^31^ (Supplementary Fig. 4). Consequently, the solid-solution strengthening effect in 3d CCAs can be rationalized by the electronegativity difference among the constituting elements, $\chi = \sqrt {\mathop {\sum }\limits_X c_X\left( {\chi _X - \left\langle \chi \right\rangle _{{\mathrm{element}}}} \right)^2}$, where *c*_X_ is the composition, (Fig. 1b) as

4 $$\sigma _{{\mathrm{SS}}} \propto \Delta \chi .$$

We use Allen’s scale as it reflects Fermi energies of d elements^32^. Additional discussion to predict = *σ*_SS_ is presented in Supplementary Note 3.

### Electronegativity difference versus ideal mixing entropy in 3d CCAs

Until now, we have assumed a random chemical mixture of elements, whereas in reality there may be some chemical short-range order among elements. Although minor, the effects of the short-range order on the yield strength would also be proportional to Δ*χ*, as (1) the short-range ordering may be favored in alloys with high Δ*χ* due to the large electronic interaction between elements, and (2) its strengthening effect is caused by the resistance to randomization of atomic configurations (breaking short-range orders), which occurs during dislocation gliding^33,34^.

**Fig. 4:**
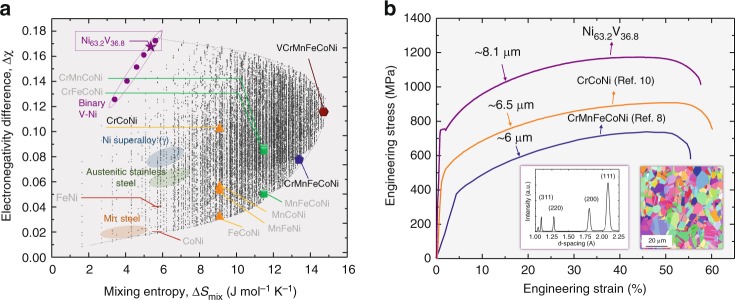
Customizing solid-solution strength in CCAs via manipulation of electronegativity difference. **a** A complexity map based on the electronegativity difference Δχ and the mixing entropy Δ*S*_mix_ for the prediction of solid-solution strengthening. All possible combinations of 3d transition metal elements (V, Cr, Mn, Fe, Co, Ni) with average VEC > 7.5 are included. The positions of the commercial alloy systems such as FeMn steel, FeNiCr steel, and γ matrix of Ni-based superalloy are shown in the diagram. Binary NiV solid-solutions are indicated in purple. **b** Tensile behavior of NiV CCA compared to various single-phase CCAs. The tensile stress-strain curves of single-phase equiatomic CrMnFeCoNi^8^ and CrCoNi^10^ are also shown here. The inset shows that NiV CCA has single fcc phase (high energy X-ray diffraction pattern) with the average grain size of 8.1 μm (Inverse pole figure map)

Figure 4a shows a diagram of the relationship between Δ*χ* and the ideal mixing entropy Δ*S*_mix_ ($= - R\mathop {\sum }\limits_X c_X{\mathrm{ln}}c_X$, where *c*_X_ is the composition of element *X* and *R* is the gas constant) for chemical complexity of 3d CCAs. We calculated Δ*χ* and Δ*S*_mix_ with all possible combinations of 3d transition metal elements (V, Cr, Mn, Fe, Co, Ni). The number of combinations is 53130 when the compositional interval is 5 at.%. We added V here due to the observation that the charge transfer is also the main contributor to atomic-level pressure in V-containing CCAs (Supplementary Fig. 5). Among these alloys, we selected alloys with VEC larger than or equal to 7.5 for their high potency of forming fcc solid-solution alloys^35^, bringing the number of configurations down to 27262. Several important CCA examples are listed in Supplementary Table 3.

There are several interesting points in diagram Fig. 4a. First, in spite of its lower chemical complexity, CrCoNi (Δ*χ* = 0.100) has a higher solid-solution strengthening than CrMnFeCoNi (Δ*χ* = 0.080). This is consistent with a previous observation that CrCoNi exhibits higher yield strength than CrMnFeCoNi^11^. Second, FeMn, basis for many commercially used iron-manganese steels, is located at a very low position (Δ*χ* ≈ 0.020). This implies good potential to develop a 3d CCA with very high strength if we optimize the plasticity mechanisms of CCA with high Δ*χ*. Third, mixing entropy, one of the key properties of HEAs (a subclass of CCAs), is not strongly correlated with the electronegativity difference and thus with high solid-solution strengthening per se. Furthermore, there is a region in which the mixing entropy should be decreased to obtain greater solid-solution strengthening effects. Mixing entropy does not include information about the difference among the constituting elements, i.e., differences in local atomic configurations. On the other hand, atomic-level pressure includes the overall information of interatomic potential energies between the central atom and the surrounding environments. Thus, the atomic-level pressure is a relevant parameter to describe the complexity of CCAs, connecting the local atomic information, such as the structure and the chemistry, to the statistical mechanical theories, and thereby to the macroscopic properties.

### Property-targeted quantitative design of 3d CCAs

In order to validate the benefits of the approach as summarized in Fig. 4a, we developed a binary NiV solid solution as a test case with the aim of achieving very high solid-solution strengthening, guided by the electronegativity difference. The eutectoid point (Ni: 63.2 at.%, V: 36.8 at.%) is chosen to obtain a single fcc phase solid solution with high stability (Supplementary Fig. 6a). Through casting, homogenization and cold rolling followed by recrystallization annealing (Supplementary Fig. 6b), we obtained a single fcc phase with the average grain size (diameter) of 8.1 µm (the inset in Fig. 4b). Figure 4b shows the representative tensile stress-strain curve of the developed NiV deformed at a strain rate of $1 \times 10^{ - 3}s^{ - 1}$ at room temperature. To show the substantial improvement in properties obtained by such a simple binary test alloy, the curves for two other CCAs (CrCoNi^10^ and CrMnFeCoNi^8^) with similar grain sizes are also presented. The yield strength of the NiV solid solution alloy is about 750 MPa and the ultimate tensile strength is close to 1200 MPa, notably outperforming the previous CCAs. Δ*χ* of NiV is 0.169, which is much larger than those of CrCoNi and CrMnFeCoNi. This clear result again confirms our hypothesis that an alloy with large electronegativity difference has a large strengthening effect, i.e., large complexity of local environments even with its relatively low mixing entropy.

## Discussion

Here we established an efficient strategy for a property-targeted quantitative atomic-level complexity design of 3d CCAs based on the atomic-level pressure approximation. In CCAs, the local atomic environment is chemically complex. For example, the element-resolved mean lattice distortions of CrMnFeCoNi HEA are small on average, namely <0.5%, but their local fluctuations (i.e., standard deviations) caused by the fluctuation in local environments are an order of magnitude larger^15^. This complexity problem is a general challenge in many disordered solutions when aiming at quantitatively predicting their properties, as it requires complicated electronic structure calculations to obtain atomically resolved unit quantities of individual atoms. We thus have devised a suite of quantitative parameters which (a) can reduce the complexity problem inherent in CCAs and HEAs; and (b) can be related to several important properties, such as the mechanical response. As a result, we demonstrated that the dominant factor for the solid-solution strengthening in 3d CCAs is the variation in the atomic-level pressures originating from charge transfer among neighboring elements. Moreover, we developed a design recipe which uses readily accessible electronegativity values to obtain the solid-solution strength in 3d CCAs, which include large sub-groups of important commercial alloys.

Prediction of the solid-solution strength does not provide a full prediction of all micromechanical strengthening mechanisms of CCAs. For instance, 2/3 of the yield strength of CrMnFeCoNi (360 MPa for the sample with a grain size of 4.4 µm) is caused by grain boundary strengthening^36^. The remaining portion of the yield strength—equivalent to its temperature-dependent portion, mainly stemming from solid-solution strengthening— is 125 MPa at room temperature. Incidentally, the atomic-level pressure is not limited to solid-solution strengthening analysis alone. Recently, a proportional relationship between the mean squared atomic displacement (and hence, lattice distortion) and grain boundary strengthening has been proposed by comparing NiCoCr and NiCoV CCAs^37^ where a significant role of grain boundary strengthening was observed for NiCoV (grain boundary strengthening: 365 MPa, temperature-dependent portion: 383 MPa). This finding shows that the atomic-level pressure (and stress) influence grain boundary strengthening as well, considering the fact that lattice distortion originates from atomic-level pressure (and stress). Furthermore, similar to solid solution strengthening, thermal activation barriers and activation volumes experienced by gliding dislocations also originate from the configurational fluctuation of the atomic-level pressure^38^. This means that the atomic-level pressure concept is a fundamental material parameter which may help understanding and quantifying also several other deformation mechanisms. Therefore, further studies are required to explore quantitative relationships between atomic-level pressure, charge transfer, and various deformation mechanisms.

Aside from the mechanical properties, the phase stability of CCAs is usually discussed in terms of mixing enthalpy and lattice strain energy of the alloy system^3^. The Miedema model^31^, a widely accepted fundamental theory to predict the mixing enthalpy, includes charge transfer as an important parameter. On the other hand, the lattice strain energy of CCAs has been discussed with regard to the difference in the atomic sizes of the constituent elements in their pure states. As the lattice strain energy also originates from the atomic-level pressure, we suggest that charge transfer dominates not only the mixing enthalpy but also the lattice strain energy, thereby affecting phase stability. Therefore, the proposed electronic-to-macro coupling approach using the concept of atomic-level pressure provides a fundamental perspective on the atomic-level complexity of disordered metallic materials. It enables computation of the local energy landscape and provides a means to condense it into theoretical models without sacrificing physics-related plausibility or capability for trend analysis, thereby helping to meet increasing needs to customize complexity-induced properties.

## Methods

### Specimen preparation

The samples were produced by arc melting method using metallurgical ingredients above 99.9% purity under Ti-gettered high-purity Argon atmosphere. The alloy button was re-melted more than five times to improve the compositional homogeneity. In cases where the alloy included Mn or Cr, the elements were placed and thus partially sealed below the other constituents to minimize evaporation.

The EXAFS samples (Ni, CoNi, FeNi, MnCoNi, MnFeNi, CrCoNi, FeCoNi, CrMnCoNi, MnFeCoNi, CrFeCoNi, CrMnFeCoNi) were then suction casted into a water-cooled copper mold with a rectangular cavity (=12 mm width × 4 mm thickness × 50 mm length). The suction casted alloys were homogenized at 1050–1200 °C for 24 h in an Ar atmosphere and eventually quenched in water. The homogenized samples underwent cold rolling to a total rolling reduction ratio of 70–85% followed by annealing above the recrystallization temperature in an Ar atmosphere followed by water quenching. Homogenization and recrystallization annealing conditions are the same as outlined in the ref. ^25^, except CrMnFeCoNi (recrystallization annealing at 1000 °C for 1 h). The bulk samples were mechanically ground into a 15 µm-thick ribbon, with SiC abrasive paper down to P4000.

The binary NiV alloy was then suction casted into a water-cooled copper mold with rectangular shape cavity (=15 mm width × 6 mm thickness × 50 mm length). The suction casted alloy was cold rolled to 30% thickness reduction to destroy the eutectoid structure established during the solidification process. The cold rolled sample was then wrapped in Ta foil and homogenized at 1075 °C for 45 h inside a quartz tube under a high vacuum condition. The homogenized sample was cold rolled to 60% thickness reduction and then underwent recrystallization annealing at 920 °C for 3 min.

### Analysis

EXAFS measurements were carried out on the 7D beamline of the Pohang Accelerator Laboratory (PLS-II, 3.0 GeV, Pohang, Korea). The measurement conditions are fully described in the ref. ^15^. The obtained datasets of 3d CCAs were aligned and processed to avoid instrumental background and absorption from other edges using the Athena in the IFEFFIT 1.2.11d suite of software^39^. All processes were conducted in the same conditions: pre-edge range with energy of −150 to −30 eV, normalization range with energy of 50–500 eV, and spline range with wave numbers of 0–11 Å^−1^. The structural parameters of the first peak were obtained through the fits of the absorption data to single scattering paths with wave numbers of 3–10.5 Å^−1^ and interatomic distances of 1–3 Å for each element (Cr, Mn, Fe, Co, Ni). In order to decrease the number of variables, we assumed (1) homogeneous compositional distribution, i.e., the coordination number of each bond type is 12/*n*, where *n* is the number of elements, and (2) uniform bond length around the fitted element (Supplementary Fig. 7a). As a result, the final average atomic radius *R*_f,solute_ of each element was obtained. The EXAFS modeling R-factors range from 0.001 to 0.003 for all fittings. The sequence of sizes of the obtained average atomic radii (Fig. 3b) matches well with the sequence of theoretical mean atomic radii (Fig. 2a, b, and Supplementary Fig. 1) obtained by DFT calculation. We also quantitatively confirmed this in^15^. The average atomic radius $\bar R_{{\mathrm{EXAFS}}} = \mathop {\sum }\limits_n c_nR_{{\mathrm{f}},{\mathrm{solute }}{\it{n}}}$ matches well with the reported average atomic radius measured using X-ray diffraction by the relation $\bar R_{{\mathrm{XRD}}} = a/\sqrt 2$ (Supplementary Fig. 7b)^23^. Although there are small differences between $\bar R_{{\mathrm{EXAFS}}}$ and $\bar R_{{\mathrm{XRD}}}$, they come from the narrow EXAFS-normalization, spline, and fitting range used here, due to the similar energy range of the elements. As the processing conditions are unified in whole samples, the small differences in the absolute values are negligible.

The phase structure of the recrystallized NiV alloy sample was identified at room temperature using high-energy X-ray diffraction (HE-XRD) performed at sector 6-ID-D of the advanced photon source (APS) at the Argonne National Laboratory, Chicago, USA. HE-XRD patterns were collected in the transmission mode. The wavelength of the X-ray beam used was 0.123686 Å and the distance between sample and 2D detector was 394.6497 mm. 2D image collected by a 2D detector was converted into 1D pattern for final data analysis using the Fit2D software.

The microstructure of the recrystallized NiV alloy was characterized using a Hitachi SU70 field emission scanning electron microscopy (SEM) with energy-dispersive X-ray spectroscopy (EDS) and electron backscattered diffraction (EBSD). The chemical uniformity was examined using EDS (X-MAX50, Horiba) at the microscopic scale. EBSD measurement was performed with a Hikari camera and the TSL OIM data-collection software. The EBSD scan step size was 75 nm and a tolerance angle of 5° was used for grain identification.

Rectangular dog-bone shaped tensile specimens, with a gauge length of 10 mm, were machined from the recrystallized sheet sample by electrical discharge machining (EDM). Oxidation layer formed during EDM cutting was removed by mechanical grinding using SiC paper. Both sides of the specimens were also ground resulting in a final thickness of ~1.6 mm and a gauge width of ~2.6 mm. Uniaxial tensile tests were carried out (Instron 5967, Instron, Norwood, USA) at an engineering strain rate of 10^-3^ s^−1^. The strain evolution during the tensile test was measured by AVE camera. In total, 5 samples were tested to confirm reproducibility.

### First-principles calculations of atomic-level pressure

Classically, the Eshelby inclusion model^40^ has been utilized to elaborate the concept of atomic-level pressure. In order to reflect atomistic and electronic effects, however, we applied here a quantum-mechanically derived atomic-level pressure approximation. The key elements that define the atomic stresses are (i) decomposition of the system’s energy into atomic contributions and (ii) observing the change of the atomic energy in response to cell distortions.

There are different approaches to decompose the energy, each of which can be used to define atomic-level stresses^41–44^. In this work we used the Voronoi tessellation as implemented in the locally self-consistent multiple scattering (LSMS) method^18,19^. In the LSMS method, the total volume is decomposed into Voronoi polyhedron around each atom and the energy is calculated within each polyhedron. The total energy of the system is then given as the sum of atomic energies. We define the energy per atom as:

$$E_i = \mathop {\smallint }\limits_{}^{{\it{\epsilon }}_{\mathrm{F}}} {\it{\epsilon }}n^i\left( {\it{\epsilon }} \right){\mathrm{d}}{\it{\epsilon }} - \mathop {\smallint }\limits_{{\mathrm{\Omega }}_i}^{} V_{{\mathrm{KS}}}\left( {\bf{r}} \right)\rho \left( {\bf{r}} \right){\bf{dr}} - \frac{1}{2}\mathop {\smallint }\limits_{{\mathrm{\Omega }}_i}^{} \rho _C\phi {\bf{dr}} + \mathop {\smallint }\limits_{{\mathrm{\Omega }}_i}^{} \rho {\bf{\epsilon }}_{{\mathbf{XC}}}\left( \rho \right){\bf{dr}} - \frac{1}{{8{\mathrm{\pi }}}}\mathop {\smallint }\nolimits^ {\boldsymbol{E}}_{{\boldsymbol{Z}}_{\boldsymbol{i}}}{\boldsymbol{E}}_{{\boldsymbol{Z}}_{\boldsymbol{i}}}{\bf{dr}},$$

where $n^i\left( {\it{\epsilon }} \right)$ is the local density of states on site *i* at energy *∈*, Ω_*i*_ is the volume of space associated with site *i*, *V*_KS_(*r*) is the Kohn–Sham potential, *ρ*(*r*) electron density, *ϕ* Poisson potential, *ρ*_C_ charge density, *∈*_XC_ local exchange correlation energy, and ${\boldsymbol{E}}_{{\boldsymbol{Z}}_{\boldsymbol{i}}}$ electrostatic field due to nuclei. The first two terms in the equation constitute site kinetic energy, the third term is the electrostatic energy due to the electronic density, the fourth term is the exchange-correlation energy and the last term is the electrostatic energy due to nuclei, each corresponding to a single atomic volume^16^. It should be noted that this energy decomposition allows full relaxation of the electron density under cell distortions minimizing the energy of the system in the spirit of the Born-Oppenheimer principle, and thus differs from the affine transformation in the scaling equations introduced by Nielsen and Martin^45,46^. The LSMS method calculates the electron density within each atomic site, and the Poisson equation is then solved with periodic boundary condition to obtain the Hartree potential. The effective Kohn-Sham potential is obtained by adding the exchange-correlation potential and the cycle is repeated until a self-consistent result is achieved. The exchange-correlation energy was treated in the local approximation using the functional of Von Barth and Hedin^47^. The atomic energy was calculated within the Voronoi polyhedral for each site. The LSMS takes into account the multiple scattering contributions from atoms only in the local interaction zone (LIZ) to obtain the electron density on that site. In our case, the LIZ radius is set at 14.8*a*_0_, and the maximum angular moment is *l* = 3.

The first principles calculation of atomic-level stresses was performed in two stages employing two different DFT based methods: (i) the projected augmented wave method (PAW)^48^ as implemented in Vienna ab-initio simulation package (VASP)^49,50^ was used for geometry optimization of the CCAs, and (ii) the LSMS method is applied to compute the atomic-level stresses as outlined above.

The disordered environment was simulated using the supercell method, based on conventional cubic special quasi-random structures (SQS)^51^ with 256 atoms included except for NiV (108 atoms). We used the plane wave energy cutoff of 400 eV and Γ-centered 2 × 2 × 2 Monkhorst-Pack grids^52^ for the Brillouin zone integration. The exchange-correlation is treated in the generalized gradient approximation (GGA), parametrized by Perdew et al.^53^ Structural optimizations were performed using the quasi-Newton algorithm and the Methfessel-Paxton smearing scheme (with smearing 0.1 eV). The equilibrium volumes of the SQS structures were first optimized till the pressure vanishes, followed by atomic relaxation until the Hellmann-Feynman forces were lower than 0.005 eV Å^−1^. The cubic cell shape was kept throughout our calculations. All supercell calculations, except for CrCoNi, were performed based on collinear magnetic states.

Next, the optimized supercell structures were subject to affine deformations, in which the volume of the supercell is changed by about 1% and atomic energies were calculated using the LSMS method. In general, the atomic-level stress tensor is defined as:^49^

$$\sigma _i^{\alpha \beta } = \frac{1}{{{\mathrm{\Omega }}_i}}\mathop {\sum }\limits_j f_{ij}^\alpha r_{ij}^\beta,$$

where *α* and *β* are Cartesian corrdinates, Ω_*i*_ is the atomic volume at site *i*, and $f_{ij}^\alpha$ and $r_{ij}^\beta$ are the force and distance between atoms *i* and *j*. The atomic-level hydrostatic pressure is then given as:

$$p_i = \frac{1}{3}{\mathrm{Tr}}\left( {\overline{\overline {\sigma _i}} } \right) = \frac{1}{3}\left( {\sigma _i^{xx} + \sigma _i^{yy} + \sigma _i^{zz}} \right)$$

Under the volume strain we applied to the supercell, the atomic-level pressure can be calculated for each atom as the negative of derivative of the energy with respect to the atomic volume. Accordingly, we calculated the energy of each atom at two different volumes and calculated the derivative by dividing the difference in energy at two different strains by the volume strain.

The electron density was integrated within the Voronoi polyhedron associated with each lattice site to calculate the local charge transfer.

## Supplementary information

Supplementary Information

## Data Availability

The data that support the findings of this study are available from the corresponding authors, at d.raabe@mpie.de, egami@utk.edu, or espark@snu.ac.kr, upon reasonable request.
