# Supplementary material for: Engineering atomic-level complexity in high-entropy and complex concentrated alloys
Source: Nat Commun. 2019 May 7;10:2090. doi: 10.1038/s41467-019-10012-7 (PMC6504951; doi:10.1038/s41467-019-10012-7)
Supplement: Supplementary file 1 — Supplementary Information [file 41467_2019_10012_MOESM1_ESM.pdf]

Supplementary Information

Engineering atomic-level complexity in high-entropy and complex concentrated alloys

Oh et al.

Supplementary Note 1

In most of the theories of CCA/HEA, the atomic size is assumed to be intrinsic to the element solute, expressed by the Goldschmidt radius ($R_{\mathrm{G,solute}}$) [1] or Pauling radius ($R_{\mathrm{P,solute}}$); the Pauling radius was derived for 12-coordinate metallic systems [2]. However, the atomic size in alloys actually depends on the environment, as shown in Fig. 3, because of the charge transfer. The steps of changing the atomic size within an alloy can be divided into 4 steps similar to the Eshelby approach:

(1) A solute atom with size $R_{\mathrm{G,solute}}$ or $R_{\mathrm{P,solute}}$ is forced into a spherical hole of slightly different size $\bar{R}$, which is the average atomic size of an alloy.

(2) The system is then relaxed to make the solute atom have 0 atomic-level pressure. After relaxation, the size of the atom becomes $R_{\mathrm{elastic,solute}}$.

(3) An atomic size further changes due to the charge transfer in the surrounding environments, maintaining its atomic-level pressure as 0. We call it zero pressure atomic size, $R_{\mathrm{zeroP,solute}}$.

(4) After full relaxation of the system, the size of the atom becomes a final atomic radius $R_{\mathrm{f,solute}}$ with atomic-level pressure P_{solute}. The average final atomic radius over the atoms of the solute element $\langle R_{\mathrm{f,solute}} \rangle$ can be measured by EXAFS, and atomic-level pressure P_{solute} can be calculated by DFT.

Considering step (4), we can estimate $R_{\mathrm{zeroP,solute}}$. During the relaxation, the atomic-level pressure of solute (=P_{solute}) gives the misfit volume strain $\varepsilon_{\mathrm{V,solute}}$,

$$\varepsilon_{\mathrm{V,solute}} = \frac{P_{\mathrm{solute}}}{B_{\mathrm{solute,cluster}}}, \qquad (1)$$

where $B_{\mathrm{solute,cluster}}$ is the average bulk modulus of a cluster of atoms including the solute atom at the center and its nearest neighboring atoms. Hence, $B_{\mathrm{solute,cluster}}$ is different from the bulk modulus of a pure element, and is similar to the bulk modulus of the alloy. Here we use the bulk modulus of the alloy B for $B_{\mathrm{solute,cluster}}$. The misfit volume strain can be described as

$$\varepsilon_{\mathrm{V,solute}} = \left(\frac{R_{\mathrm{zeroP,solute}}}{R_{\mathrm{f,solute}}}\right)^3 - 1. \qquad (2)$$

Hence, the two equations yield,

$$R_{\text{zeroP,solute}} = R_{\text{f,solute}} \cdot \left(1 + \frac{P_{\text{solute}}}{B}\right)^{1/3}. \quad (3)$$

Therefore, the misfit volume should be defined by the atomic-level pressure, and thus by the difference between $R_{\text{zeroP,solute}}$ and $R_{\text{f,solute}}$ to reflect the electronic effects (using the relation $V = 4\sqrt{2}R^3$). However, in the literature, the misfit volume of element n is often derived from the lattice parameter data as $1/\bar{R}_{\text{XRD}}\,(\partial\bar{R}_{\text{XRD}}/\partial c)_n$, where c is the composition, following the way of defining the size misfit factor in dilute alloys [3,4] using XRD. This approach assumes the consistent atomic size (or negligible change) at the interested composition range. As shown in Supplementary Fig. 7b, the average atomic radius $\bar{R}_{\text{EXAFS}}$ is approximately equal to the average atomic radius measured by XRD $\bar{R}_{\text{XRD}}$. This implies that, assuming the consistent atomic size, $1/\bar{R}_{\text{XRD}}\,(\partial\bar{R}_{\text{XRD}}/\partial c)_n$ is equivalent to $\bar{R}_{\text{EXAFS}} - \langle R_{\text{f,solute}}\rangle_n$, and the total deviation in the misfit volume strain becomes $3\,\Delta(\langle R_{\text{f,solute}}\rangle)/\bar{R}_{\text{EXAFS}}$. As a result, the calculated σ_{SS} values, plugging $\langle R_{\text{f,solute}}\rangle$ values together with reported v and μ values [5] into the equation 15 in ref. 6, are about 30 times smaller than the measured values (Supplementary Table 1). This shows the inapplicability of the previous XRD-based approach for measuring the misfit volume of the present 3d CCAs.

As the Poisson's ratio of 3d CCAs is usually close to 1/3, B can be approximated to $\mu(1+v)/(1-v)$, where μ is the shear modulus, and v is the Poisson's ratio. Equation (1) in the manuscript can be directly derived from equation 15 in ref. 6 from the definition of the misfit volume as

$$\sigma_{\text{SS}} = 3.06 \times 0.051 \times f_1(\omega_c) \times (\alpha\mu)^{-1/3} \times \Delta P_{\text{solute}}{}^{4/3} \times 0.63, \quad (4)$$

where $f_1(\omega_c)$ is the core coefficient ~0.35. The calculated σ_{SS} values with P_{solute} are about 4 times larger than the measured values (Supplementary Table 1). The difference between calculated and measured strengths reduces with the present definition of the misfit volume. The remaining difference may come from the dislocation substructure or other dislocation interaction parameters, hence further studies are required to this end. However, as mentioned, the exact scaling is not the main focus of this study because the proportional relationship of solid-solution strengthening and 4/3 power of ΔP_{solute} should always hold.

Supplementary Note 2

From the computational results, $\Delta(dQ)$ can be calculated as

$$\Delta^2(dQ) = \frac{1}{N}\sum_{i=1}^{N}(dQ_i - \langle dQ\rangle)^2, \qquad (5)$$

where N is the number of atoms, dQ_i is the charge transfer of the i-th atom, and $\langle dQ\rangle$ is the average of the atomic charge transfers over all the atoms calculated as

$$\langle dQ\rangle = \frac{1}{N}\sum_{i=1}^{N} dQ_i. \quad (6)$$

To understand the impact of each chemical element in more detail, we consider decomposing $\Delta(dQ)$ using the law of total variance as,

$$\Delta^2(dQ) = \Delta^2_{\text{element}}(\langle dQ\rangle_X) + \langle \Delta^2_X(dQ)\rangle_{\text{element}}. \qquad (7)$$

The symbol $\langle dQ\rangle_X$ denotes the average of the charge transfers over the atoms of the element X as

$$\langle dQ\rangle_X \equiv \frac{1}{|S_X|}\sum_{i\in S_X} dQ_i, \qquad (8)$$

where the set S_X contains the indices of the atoms of the element X. Note that S_X satisfies

$$N = \sum_X |S_X|. \quad (9)$$

The symbol $\Delta^2_X(dQ)$ denotes the variance of the charge transfers for the element X which is calculated as

$$\Delta^2_X(dQ) = \frac{1}{|S_X|}\sum_{i\in S_X}(dQ_i - \langle dQ\rangle_X)^2. \qquad (10)$$

The symbol $\langle\cdots\rangle_{\text{element}}$ denotes the weighted average of an element-specific quantity v_X over the element as

$$\langle v\rangle_{\text{element}} = \frac{1}{N}\sum_X |S_X| v_X. \qquad (11)$$

The symbol $\Delta^2_{\text{element}}(v)$ is the weighted variance of v_X among the constituent elements defined as

$$\Delta^2_{\text{element}}(v) \equiv \frac{1}{N}\sum_X |S_X|(v_X - \langle v\rangle_{\text{element}})^2. \quad (12)$$

Thus, the term $\Delta^2_{\text{element}}(\langle dQ\rangle_X)$ denotes the macroscopic difference in charge transfer between consisting elements, while the term $\langle \Delta^2_X(dQ)\rangle_{\text{element}}$ represents the variance in charge transfer of the atoms of the same elements, which is caused by different local chemical environments.

Supplementary Note 3

Strictly speaking, σ_{SS} depends not only on $\Delta\chi$ but also on shear modulus due to the line tension (equation (1) in the manuscript). However, the shear moduli of 3d CCAs are quite similar [5], and we neglect this dependency for the sake of simplicity. There is another effect of the modulus on the solid-solution strength, which is the modulus misfit effect. However, the model proposed in ref. 6 does not consider the modulus misfit effect, as it is a minor effect in *fcc* alloys, where the edge components of the dislocations dominate the solid-solution strengthening [7]. It is also known that σ_{SS} of an *fcc* alloy can be affected by the dislocation dissociation distance if it is ten times shorter than the Burgers vector; in such cases the corresponding stacking fault energies (SFEs) are larger than 100 mJ m^{-2} [6]. However, most 3d CCAs show much lower stability of *fcc* phase, i.e. also smaller SFE as compared to pure Ni whose SFE is about 120–130 mJ m^{-2} [8]. σ_{SS} of 3d CCAs, thus, is independent to the dissociation of dislocation except low alloyed Ni alloys, which in any case have small solid-solution strengthening. As a consequence, we can broadly predict σ_{SS} of 3d CCAs by a linear fitting of σ_{SS} and $\Delta\chi$ in Fig. 3d as

$$\sigma_{SS} = (4293 \pm 448)\Delta\chi + (84 \pm 27)\ (\text{MPa}). \qquad (13)$$

The temperature-dependent part of the yield strength of Ni at 0 K was measured as 46 MPa. Major part of the extra term, 84 ±27 MPa, is thus expected to come from the Peierls friction stress.

Supplementary Table 1 | Experimentally measured zero-temperature flow stresses of 3d CCAs given by ref. 5. As Ni is not a solid solution, the zero-temperature stress value of Ni is the Peierls friction stress. We assumed that the friction stress would be small and invariant depending on alloy compositions compared to solid-solution strengthening. We thus regarded the value of Ni as a reference point. Calculated solid-solution strengths using final atomic radius σ_{SS} ($R_{f,solute}$) and DFT calculated atomic-level pressure σ_{SS} (ΔP_{solute}) are also listed (see Supplementary Note 1).

	σ_{SS} (measured) (MPa)	σ_{SS} ($R_{f,solute}$) (MPa)	σ_{SS} (ΔP_{solute}) (MPa)
Ni	46	0	0
CoNi	130	0	420
FeNi	341	17	876
MnCoNi	302	29	1455
MnFeNi	283	22	1557
CrCoNi	489	11	1810
FeCoNi	292	11	689
CrMnCoNi	491	26	1679
MnFeCoNi	282	17	1348
CrFeCoNi	443	11	1552
CrMnFeCoNi	423	19	1493

Supplementary Table 2 | Elemental pieces of atomic information used in present research; Electronegativity [9]; Valence electron concentration [10]; Metallic radius in Pauling scale (coordination number=12) [11].

	V	Cr	Mn	Fe	Co	Ni
Electronegativity, χ_{Allen}	1.53	1.65	1.75	1.80	1.84	1.88
Valence electron concentration, VEC (e)	5	6	7	8	9	10
Metallic radius, $R_{\mathrm{P,solute}}$ (Å)	1.338	1.267	1.261	1.260	1.252	1.244

Supplementary Table 3 | Examples of the combinations of 3d elements (V, Cr, Mn, Fe, Co, Ni) and the resultant $\Delta\chi$, ΔS_{mix}, and VEC.

	Description	V	Cr	Mn	Fe	Co	Ni	$\Delta\chi$	ΔS_{mix} (J·mol^{-1}·K^{-1})	VEC (e)
1	CoNi	0	0	0	0	50	50	0.020	5.76	9.5
2	FeNi	0	0	0	50	0	50	0.040	5.76	9.0
3	MnCoNi	0	0	33.3	0	33.3	33.3	0.054	9.13	8.6
4	MnFeNi	0	0	33.3	0	0	33.3	0.054	9.13	8.3
5	CrCoNi	0	33.3	0	0	33.3	33.3	0.100	9.13	8.3
6	FeCoNi	0	0	0	33.3	33.3	33.3	0.033	9.13	9.0
7	CrMnCoNi	0	25	25	0	25	25	0.089	11.53	8
8	MnFeCoNi	0	0	25	25	25	25	0.048	11.53	8.5
9	CrFeCoNi	0	25	0	25	25	25	0.087	11.53	8.25
10	CrMnFeCoNi	0	20	20	20	20	20	0.080	13.38	8
11	VCrMnFeCoNi	16.6	16.6	16.6	16.6	16.6	16.6	0.119	14.87	7.5
12	γ matrix Ni-superalloy	0	20	0	20	0	60	0.090	7.90	8.8
13	Austenitic stainless steel	0	20	0	70	0	10	0.068	6.67	7.8
14	Manganese steel	0	0	35	65	0	0	0.024	5.38	7.7
15	$Ni_{63.2}V_{36.8}$ (present)	36.8	0	0	0	0	63.2	0.167	5.46	8.2

Supplementary Figure 1 | Schematic illustration of atomic-level pressure. Classical mechanistic point of view: Atomic-level pressure is described through the Eshelby back-stress effect; energy perspective: Atomic-level pressure reflects the average properties of interatomic potential between a center atom and surrounding atoms.

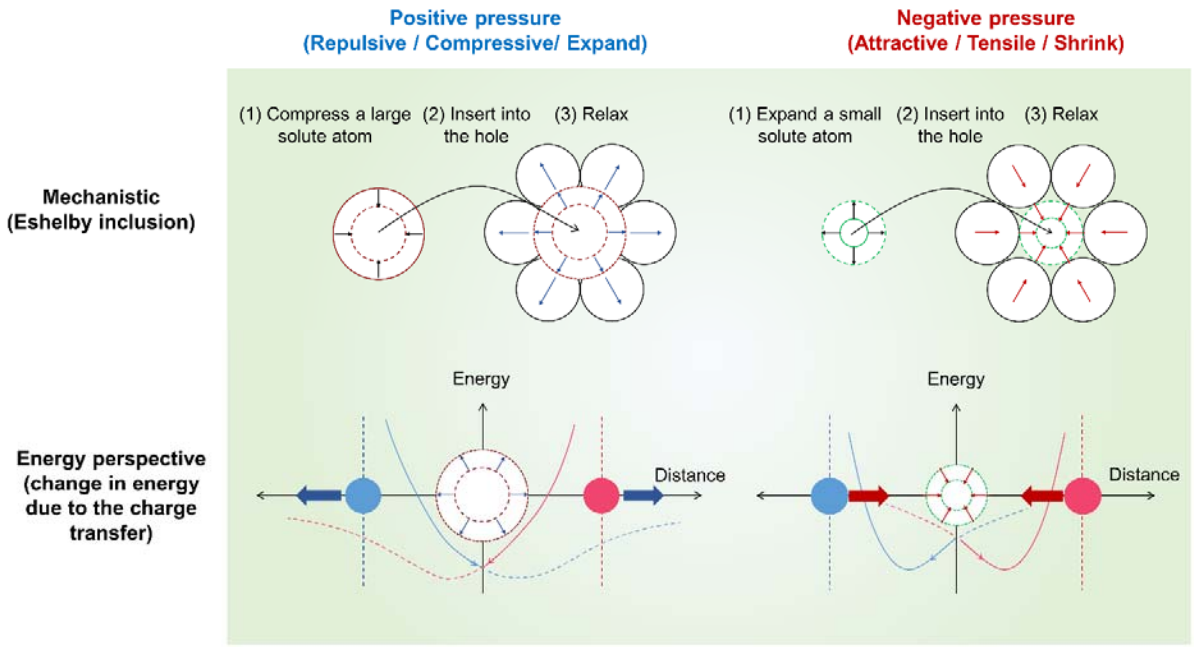

Supplementary Figure 2 | Atomic-level pressure versus atomic volume relation in 3d CCAs including a, CoNi, **b**, MnCoNi, **c**, MnFeNi, **d**, CrCoNi, **e**, FeCoNi, **f**, CrMnCoNi, **g**, MnFeCoNi, and **h**, CrFeCoNi.

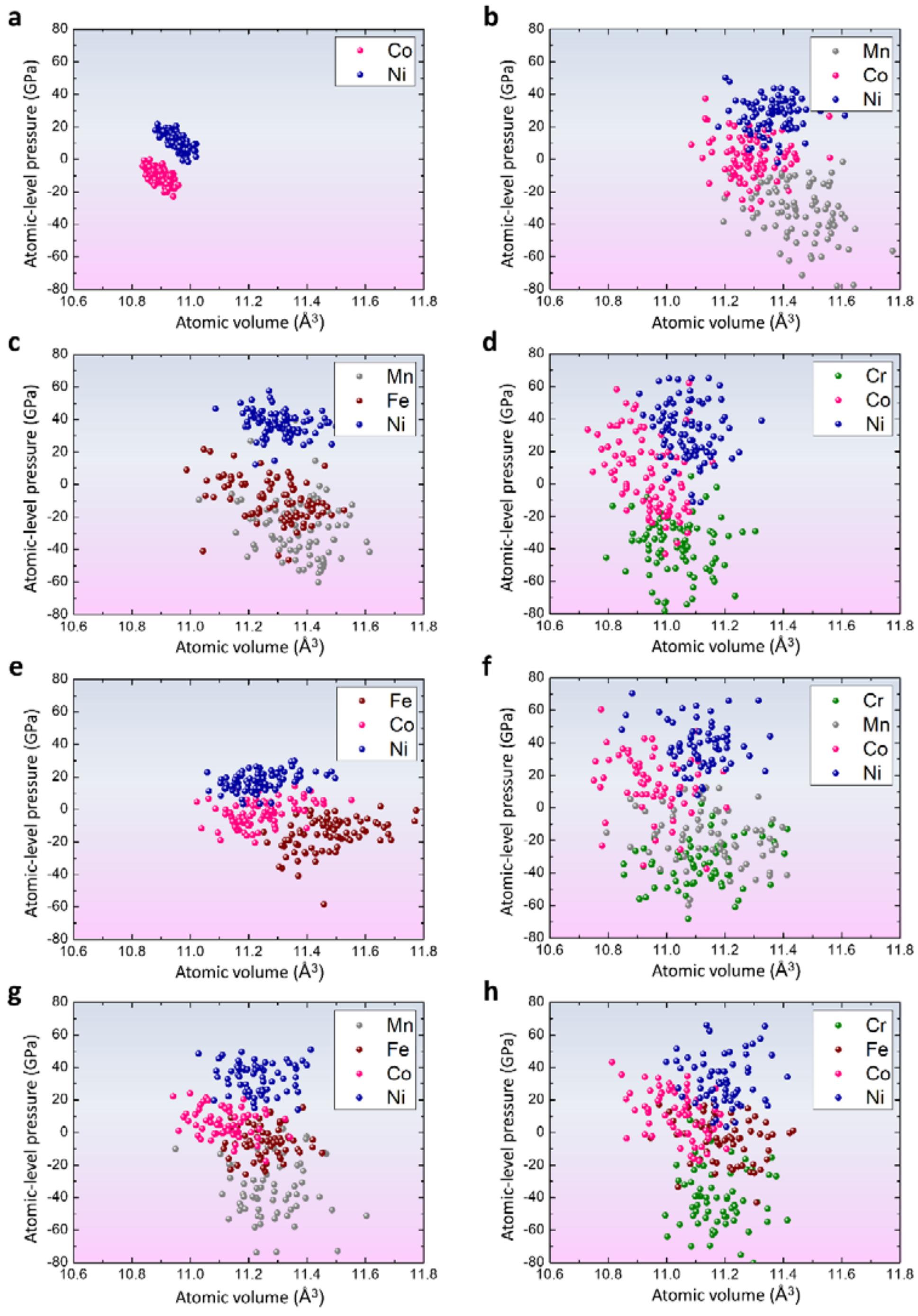

Supplementary Figure 3 | Complexity diagram established based on the Pythagorean relation between macroscopic $\Delta_{\text{element}}(\langle dQ\rangle_X)$ (pink), configurational $\sqrt{\langle\Delta_X^2(dQ)\rangle_{\text{element}}}$ (teal), and total $\Delta(dQ)$ (black) difference for the charge transfer in equation (3). The length of each line represents the size of deviation in charge transfer. The similarity of the triangles thus reflects the consistency of the relation between macroscopic and configurational difference of charge transfer in the 3d CCAs.

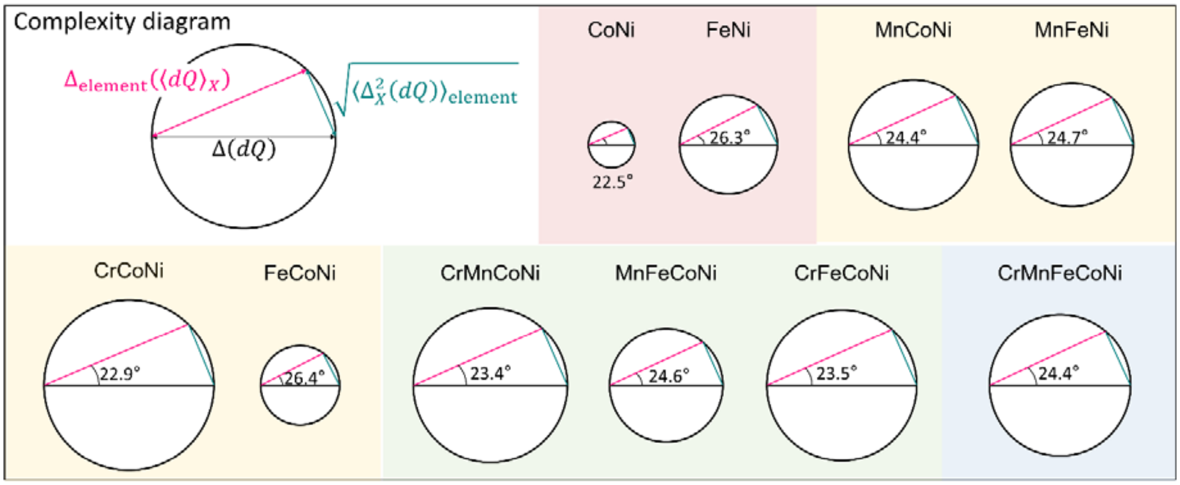

Supplementary Figure 4 | Approximation of the charge transfer using the electronegativity difference. DFT calculated average charge transfer $\langle dQ \rangle_X$ and local electronegativity difference $\chi_X - \langle \chi \rangle_{\mathrm{element}}$ of each element show a good linear relation.

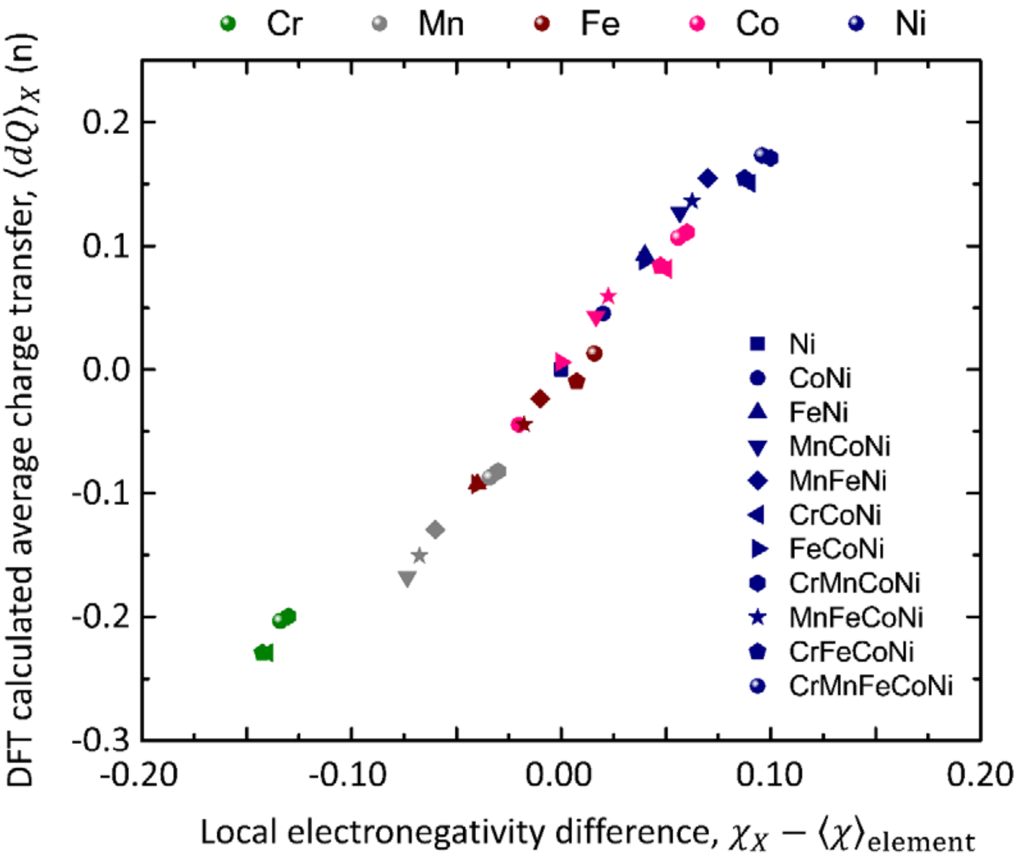

Supplementary Figure 5 | Relationship between atomic-level pressure and the charge transfer of binary NiV solid solution. The model contains 108 atoms in a supercell with 69 Ni atoms and 39 V atoms.

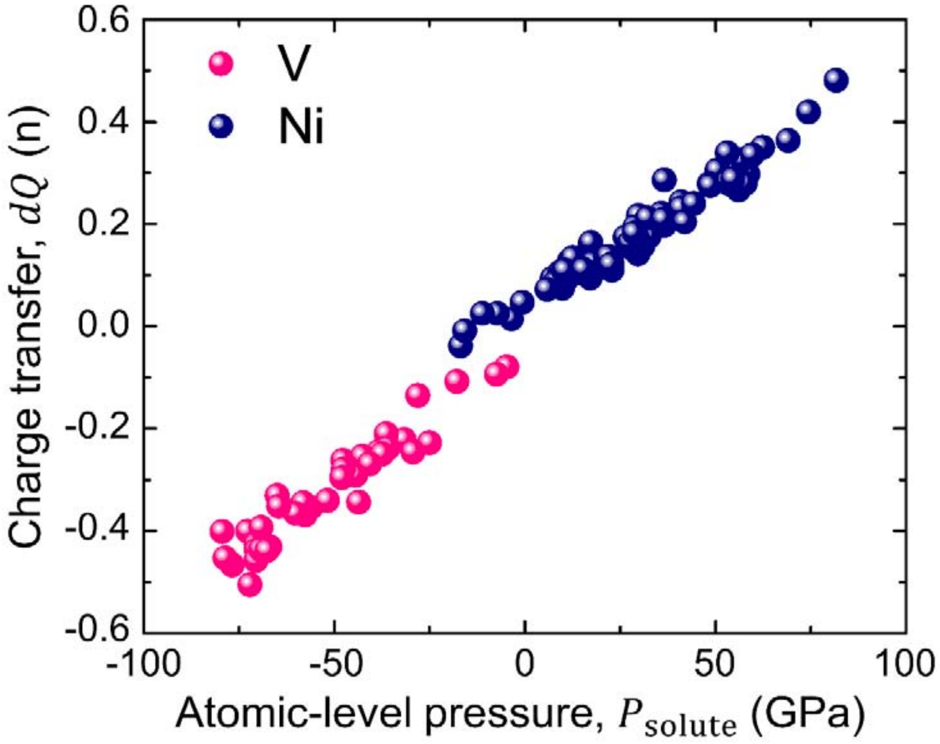

Supplementary Figure 6 | a, V-Ni binary phase diagram calculated using the Thermocalc approach (database: SSOL6), **b**, SEM images showing the microstructure, and EDS results showing the homogeneous distribution of elements; **c**, V Ka; **d**, Ni Ka.

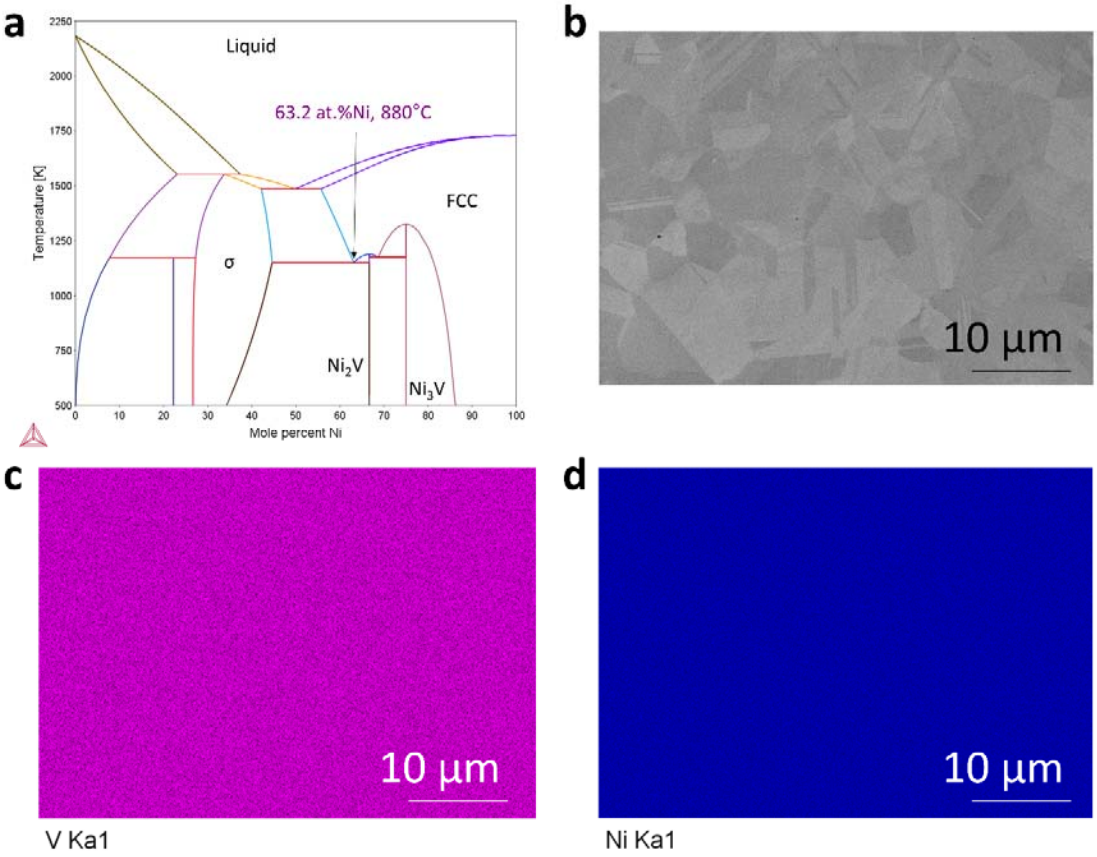

Supplementary Figure 7 | EXAFS fitting process. **a**, Fourier transform profiles of the experimental EXAFS spectra and the fitted results of Ni K-edge of CrMnFeCoNi, **b**, Comparison between average atomic radii of the present 3d CCAs measured by EXAFS and average atomic radii measured by XRD.

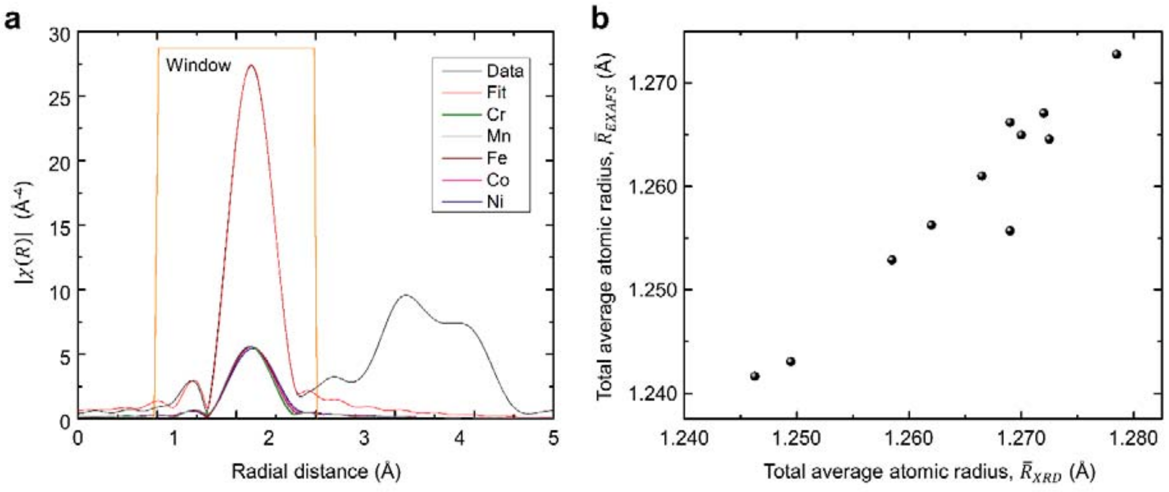

Supplementary References

1. Goldschmidt, V. M. Uber atomabstande in metallen. *Z. Phys. Chem.* **133,** 397–419 (1928).
2. Pauling, L. Atomic radii and interatomic distances in metals. *J. Am. Chem. Soc.* **69,** 542–553 (1947).
3. Lubarda, V. On the effective lattice parameter of binary alloys. *Mech. Mater.* **35,** 53–68 (2003).
4. Uesugi, T. & Higashi, K. First-principles studies on lattice constants and local lattice distortions in solid solution aluminum alloys. *Comput. Mater. Sci.* **67,** 1–10 (2013).
5. Wu, Z., Bei, H., Pharr, G. M. & George, E. P. Temperature dependence of the mechanical properties of equiatomic solid solution alloys with face-centered cubic crystal structures. *Acta Mater.* **81,** 428–441 (2014).
6. Varvenne, C., Luque, A. & Curtin, W. A. Theory of strengthening in fcc high entropy alloys. *Acta Mater.* **118,** 164–176 (2016).
7. Argon, A. S. Strengthening Mechanisms in Crystal Plasticity (Oxford Univ. Press, 2004).
8. Carter, C. B. & Holmes, S. M. The stacking-fault energy of nickel. *Phil. Mag.* **35,** 1161–1172 (1977).
9. Mann, J. B., Meek, T. L., Knight, E. T., Capitani, J. F. & Allen, L. C. Configuration energies of the d-Block elements. *J. Am. Chem. Soc.* **122,** 5132–5137 (2000).
10. Guo, S., Ng, C., Lu, J. & Liu, C. T. Effect of valence electron concentration on stability of fcc or bcc phase in high entropy alloys. *J. Appl. Phys*. **109,** 103505 (2011).
11. Pauling, L. Atomic radii and interatomic distances in metals. *J. Am. Chem. Soc.* **69,** 542–553 (1947).
